# Trend in Ambient Ozone and an Attempt to Detect Its Effect on Biota in Forest Ecosystem. Step I of Lithuanian Studies

**DOI:** 10.1100/tsw.2007.53

**Published:** 2007-03-21

**Authors:** Algirdas Augustaitis, Ingrida Augustaitiene, Almantas Kliucius, Gintautas Mozgeris, Gintaras Pivoras, Rasele Girgzdiene, Kestutis Arbaciauskas, Irena Eitminaviciute, Reda Mazeikyte

**Affiliations:** ^1^ Lithuanian University of Agriculture, LT-53362 Kaunas dstr., Lithuania; ^2^ Institute of Physics, Savanoriu 231, LT-02300, Vilnius, Lithuania; ^3^ Vilnius University, Institute of Ecology, LT-08412, Vilnius, Lithuania

**Keywords:** ambient ozone, tree crown defoliation, soil microarthropods, stream macroinvertebrates, small mammals

## Abstract

The presented study aimed to explore the relationships between ambient ozone (O_3_) and tree defoliation, specific diversity, and abundance of soil microarthropods, stream macroinvertebrates, and small mammals (mainly rodents) in order to test the hypothesis that changes in the considered objects of the forest ecosystem could be related to changes in ambient O_3_, concentration of which is below critical level. The observations were carried out from 1994 at three integrated monitoring stations. The obtained data revealed that only peak O_3_ concentrations (from 125–215 μg·m^-3^) had significant effect on changes in the considered components of forest biota.

## INTRODUCTION

Ozone ($O_3$) air pollution has been recognized as a major phytotoxic agent in North America and Europe since the middle of the last century[1,2,3,4,5,6,7]. In contrast to $SO_2$, the continuing rise in the emissions of precursor substances (NO, $NO_2$)[8,9] resulted in a rise in $O_3$ concentrations, which occurred on a large scale over the past century[10,11,12,13]. This increase in $O_3$ concentrations and their adverse effects have become a considerable concern[14,15,16,17,18].

Ambient $O_3$ may interrupt cell membrane function that, in turn, will affect metabolic activity in other parts of the cell, either plant or animal[19]. The phytotoxic effect of ambient $O_3$ on plants is well known. Over a long time, its low doses affect mainly physiological processes and metabolism[20], while short periods of high doses not only cause visible injuries[21], but also result in growth reduction[3,4]. However, our knowledge of potential long-term $O_3$ impacts on perennial plant species or their communities under natural conditions, especially in northern Europe, is limited[22].

Negative $O_3$ effect on some terrestrial arthropods is known[23,24,25,26,27]. It occurs through one or more of the following: (1) changes in litter quality and quantity, (2) decreased carbon allocation to roots,

(3) altered root exudation and soil carbon dioxide ($CO_2$) flux, and (4) possibly decreased root growth and increased root mortality[27,29]. Additional effects may occur through increase in availability of a key nutrient, nitrogen, which is critical for arthropod growth[23,30,31]. However, since $O_3$ is unlikely to penetrate the soil, its direct impact is hardly plausible in real-world situations[28].

Mammals are considered to be the highest form of life in the forest ecosystem. However, under elevated exposure, $O_3$ markedly affects their lung tissue[32,33] and several hematological parameters[34] including decreases in red blood cells[35] and an increase in the possibility of death[19,36,37]. On the regional scale, relationships between $O_3$ exposure, and diversity and abundance of small mammals, could be useful for analyzing causative effect of $O_3$ on forest ecosystems in general.

In Lithuania, on the coastal part of the country, the continuous measurements of $O_3$ concentrations, with the aim of monitoring and assessing $O_3$ and other pollutants, started in 1980 at the EMEP station Preila[38]. In 1994–95, observations of $O_3$ concentrations were expanded to three new stations established in national parks, according to the Integrated Monitoring program[39]. The aim of this program was to determine the long-term state of a terrestrial ecosystem and its changes, with respect to the regional variation and impact of air pollutants, including ambient $O_3$, on biota. The obtained data indicated that the concentration of $O_3$, among the concentrations of the other air pollutants ($SO_2$, $NO_3^-$ and $NH_4^+$), and N and S deposition, reached the levels that were potentially phytotoxic for forests. However, in 2002, the first visible $O_3$-induced injury was observed within the framework of the ICP Forest Monitoring Program, level I[40]. After a few years, significant relationships between acidifying compounds and different components of biota[41], including pine defoliation[42,43], were derived. These initial results initiated more thorough studies of the stress-response relationship in forests under the regional pollution load, which has recently become of the greatest concern.

In three-step studies, we analyze the impact of the ambient $O_3$ by employing extensive (analysis of the $O_3$ impact on station-wise mean value of the different components of forest biota, from the lowest to the highest life forms, with small number of degrees of freedom on catchments of IMS) and intensive (analysis of the $O_3$ impact on pine stand condition and growth with high number of degrees of freedom on additional data set from net of local pine stand monitoring) methods. In the first step of our studies, we tested a hypothesis that temporal and spatial changes in station-wise mean value of tree defoliation, abundance, and specific diversity of soil microarthropods, stream macroinvertebrates, and small mammals in natural forest ecosystems are related to changes in ambient $O_3$ concentrations. To test that hypothesis, correlative coefficients of different $O_3$ concentrations and station-wise mean values of the different components of biota were compared. That was done to find the component of biota most sensitive to $O_3$, and the most acceptable concentration or index of $O_3$ exposures to be used in the more thorough studies.

## METHOD AND MATERIALS

The study was based on the observed data available from three Integrated Monitoring Stations (IMS) established in Aukstaitija (LT-01) and Dzukija (LT-02) National Parks (NPs) in 1994 and Zemaitija (LT-03) NP in 1995 (Fig. 1). In 2000, due to limited funding, the LT-02 station was closed and the monitoring terminated. Therefore, only the data on pollution and considered components of biota for the period 1994–1999 were used for this study.

Tree crown condition was assessed using the methodology of the ICP Forest Monitoring Program[44]. More than 1000 trees on 50 permanent observation plots (POPs) at LT-01, 1200 trees on 58 POPs at LT-02, and 600 trees on 37 POPs at LT-03 located in IMS basins were monitored annually from the end of August through the beginning of September. Based on these data, mean annual defoliation of the considered tree species per IMS was computed.

The diversity and abundance of soil microarthropods, small mammals, and stream macroinvertebrates were examined according to the Manual for Integrated Monitoring[39]. Station-wise mean annual values were computed for each IMS.

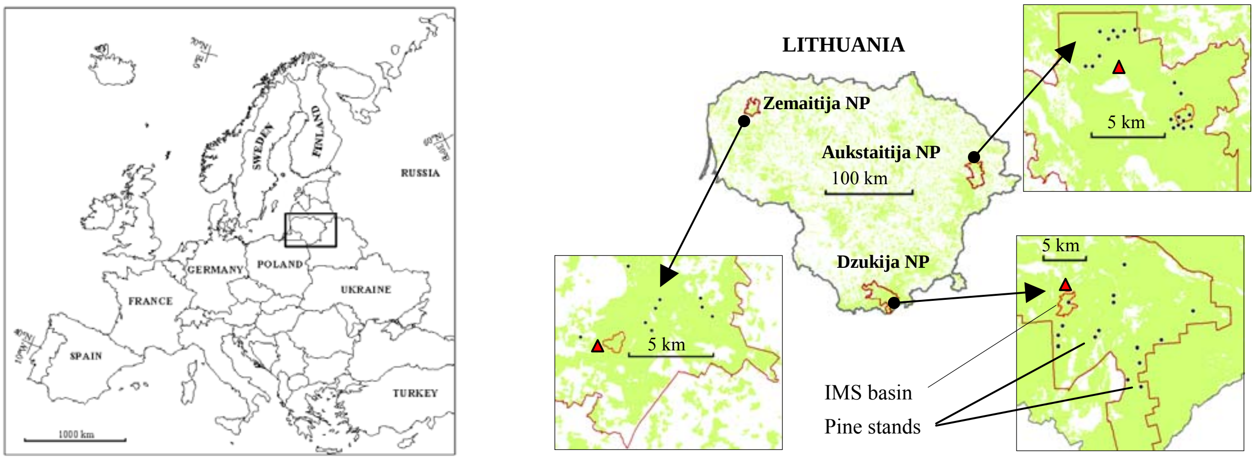

**FIGURE 1.** Location of Aukstaitija, Dzukija, and Zemaitija IMS (▲) basins with POPs and permanent observation pine stands in Lithuanian NPs.

Ozone concentrations were measured continuously using commercial UV-absorption monitors $O_3$ 41M (Environment S.A., France) and ML9811 (Monitor Labs) with an air inlet at the height of 2.5 m above ground. The instruments were calibrated periodically every year. Hourly data on peak $O_3$ value, their annual average, and average from April through August were used in the analysis. AOT40 values, which define the potential risk of $O_3$ for vegetation[45], were calculated according to the requirements of the 2002/3/EC directive. For crops, the critical level is set to 3.0 ppmh (AOT40-1); for forest trees, 10 ppmh (AOT40-2)[46]. Exceedance of the AOT40-2 threshold would indicate a risk of tree biomass loss of more than 10%[47].

The possible effect of ambient $O_3$ on forest ecosystems was analyzed in a two-step statistical analysis. First, a correlative analysis of different concentrations of $O_3$ and station-wise means of the considered response variables was performed. Degree of freedom in the step I of the study reached 28, when annual data represented all stations over the observation period, and smaller, when data represented one or two stations. At the next stage, using multiple regression analysis, we attempted to quantify the contribution of the most significant $O_3$ index to the integrated impact of natural (air temperature and precipitation amount) and anthropogenic (air concentration of acidifying compounds, their concentration in precipitation and deposition) factors on defoliation and growth of Scots pine trees. Degree of freedom for defoliation analysis ($n = 421$) and growth analysis ($n = 102$) enabled us to meet the objectives of the second and third steps of the presented studies.

## RESULTS

### Trend in Ambient $O_3$ Level in Forest Ecosystems

Ozone concentration data at IMS show no clear trend in temporal changes in the annual mean, and mean values from April through August as well as in the AOT40. However, decline in the peak concentrations from 215 to 125 $\mu g \cdot m^{-3}$ was observed until 2001 (Fig. 2). After 2001, no significant increase in both means (annual and April–August period) and peak concentrations was observed. The peak hourly $O_3$ concentrations varied from 125 to 165 $\mu g \cdot m^{-3}$ during the summer period and were typical for other parts of Central Europe[7,48,49,50]. High (more than 120 $\mu g \cdot m^{-3}$) for Lithuanian conditions, $O_3$ concentrations are mostly observed when air masses were transported from Western Europe.

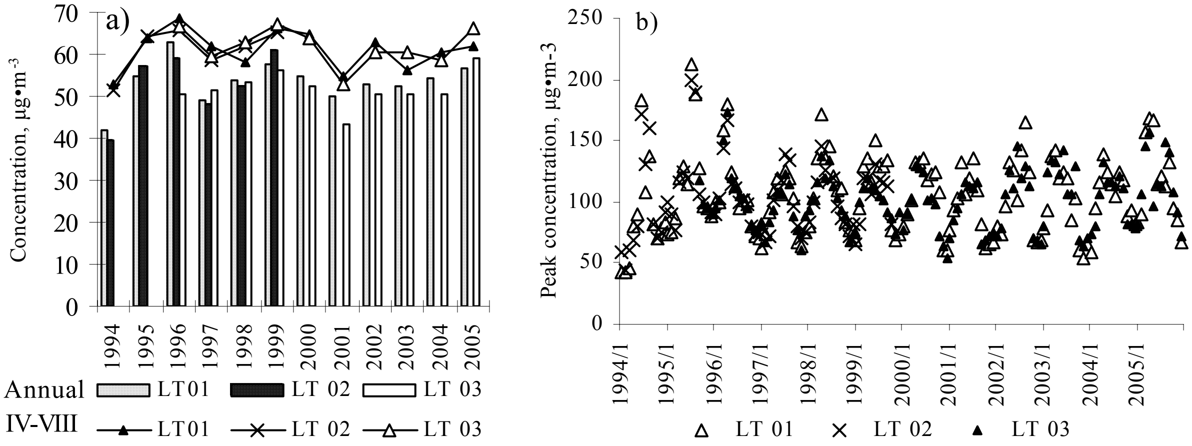

**FIGURE 2.** The variation in $O_3$ annual and April-August period mean (a) and peak concentrations (b)

The $O_3$ levels were rather similar at all stations (Fig. 2), whereas the differences in AOT40 were more significant because AOT40 values were calculated only during the daytime, when $O_3$ concentrations were higher than 80 $\mu g{\cdot}m^{-3}$. Such $O_3$ levels were mostly observed on sunny days, the number of which differed significantly between the western (LT-03) and eastern parts (LT-01) of Lithuania, or during the $O_3$ transport from the polluted regions. The computed AOT40 values for the protection of forest at LT-01 and LT-02 ranged from 8000–21,000 $\mu g{\cdot}m^{-3}$ h while at LT-03 only from 5000–12,000 $\mu g{\cdot}m^{-3}$ h.

The concentration of $O_3$ among the concentrations of the other monitored air pollutants ($SO_2$, $\Sigma NO_3^-$ and $\Sigma NH_4^+$) reached the closest to critical phytotoxic level. The AOT40 value for the protection of vegetation (6000 $\mu g{\cdot}m^{-3}$ h) was exceeded at all stations for almost all considered years[51]. The critical level 20,000 $\mu g{\cdot}m^{-3}$ h for the protection of the forest was observed only at LT-01 in the year 1999. The highest peak $O_3$ value, 213 $\mu g{\cdot}m^{-3}$, was observed only in the year 1995, while higher than 160 $\mu g{\cdot}m^{-3}$ value, at the beginning of observation and recently, in the years 2002 and 2005. Peak $O_3$ concentrations most often were observed in spring, i.e., in April and May (Fig 2b).

## Relationships between $O_3$ and Considered Components of Forest Biota

### *Tree Crown Defoliation*

In 1996, the highest level of mean defoliation was observed: at LT-01, 30.7 ± 0.7%; LT-02, 35.6 ± 0.9%; and LT-03, 26.4 ± 0.9%. Afterwards, until 2001, a significant decrease in defoliation was observed (at LT-01 up to 23.2 ± 0.4%; LT-02, 30.0 ± 0.8%; LT-03, 20.3 ± 0.6%). Between 2001 and 2005, tree crown defoliation at LT-03 gradually increased up to 24.9 ± 0.9 %, whereas at LT-01 defoliation was stable at the level of 23.1 ± 0.6%. Condition of Scots pine and birch (*Betula pendula 'Crispa'* and *B. pubescens Ehrh.*) was better than that of Norway spruce (*Picea abies Karst.*). The peak value of $O_3$ concentrations had significant impact on changes in birch and pine defoliation (Table 1). Mean defoliation of the considered tree species was more related to the AOT40 index for forest than crop vegetation; however, these relationships in most cases were not significant ($p > 0.05$).

### Diversity and Abundance of Soil Pedobionts

The common trend of changes in abundance and specific diversity of soil pedobionts was not statistically significant; however, the increase in microarthropod diversity between 1995 and 2001 resulted in significant changes in the mineralization-humification (M/H) ratio process[41].

**TABLE 1**
**Relationships between Ambient $O_3$ and Defoliation of Considered Tree Species on IMS Territories over 1994–2005 and Their Significance**

| Species | Main Statistics | Mean Value | | Peak Value | AOT40 Value |
|---|---|---|---|---|---|
| | | Annual | IV–VIII | | |
| *Quercus robur* (n = 9) | r | 0.647 | 0.518 | 0.123 | 0.527 |
| | *p* < | 0.060 | 0.153 | 0.753 | 0.180 |
| *Alnus glutinosa* (n = 15) | r | –0.157 | –0.318 | –0.323 | –0.398 |
| | *p* < | 0.576 | 0.248 | 0.241 | 0.200 |
| *Betula* spp. (n = 25) | r | –0.268 | –0.189 | 0.465 | 0.181 |
| | *p* < | 0.196 | 0.365 | 0.019 | 0.431 |
| *Populus tremula* (n = 12) | r | –0.084 | 0.070 | 0.366 | 0.379 |
| | *p* < | 0.795 | 0.829 | 0.241 | 0.225 |
| *Picea abies* (n = 25) | r | –0.260 | –0.077 | 0.353 | 0.510 |
| | *p* < | 0.210 | 0.716 | 0.084 | 0.018 |
| *Pinus sylvestris* (n = 28) | r | –0.142 | –0.122 | 0.549 | 0.077 |
| | *p* < | 0.471 | 0.538 | 0.002 | 0.740 |

Relationships among different $O_3$ indices and the considered microarthropod parameters revealed that peak $O_3$ concentrations demonstrated more significant relationships with microarthropod diversity than their abundance (Fig. 3). The strongest relationships were established between peak $O_3$ concentrations and *Oribatidae, Acaridae,* and M/H ratio, what most likely indicated their susceptibility to $O_3$ exposure.

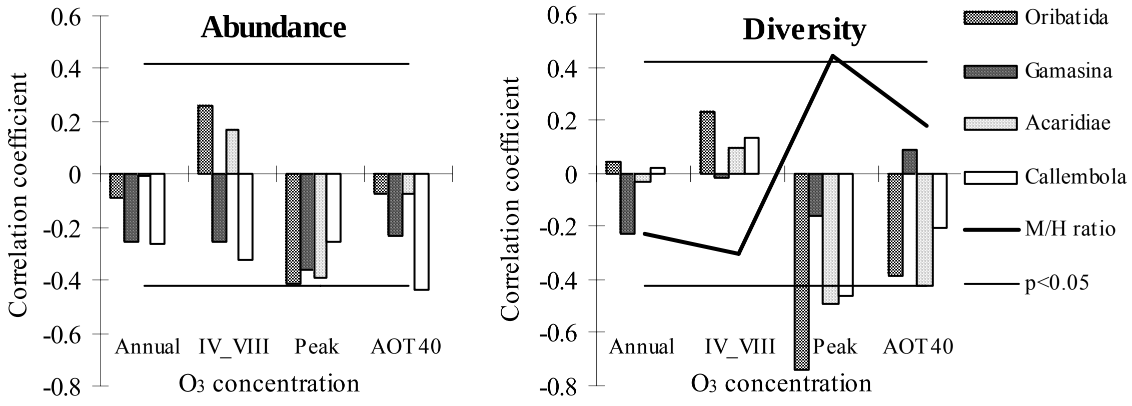

**FIGURE 3.** Relationships among ambient $O_3$ and soil arthropod abundance, diversity, and M/H ratio.

### Diversity and Abundance of Small Mammals

There was no significant trend in changes in species number and mammal abundance. However, peak in both diversity and abundance was recorded in 1998–99[41]. Analysis of the correlation coefficients showed that only relationship between peak $O_3$ concentration and diversity of small mammals was significant ($p < 0.05$) (Fig. 4).

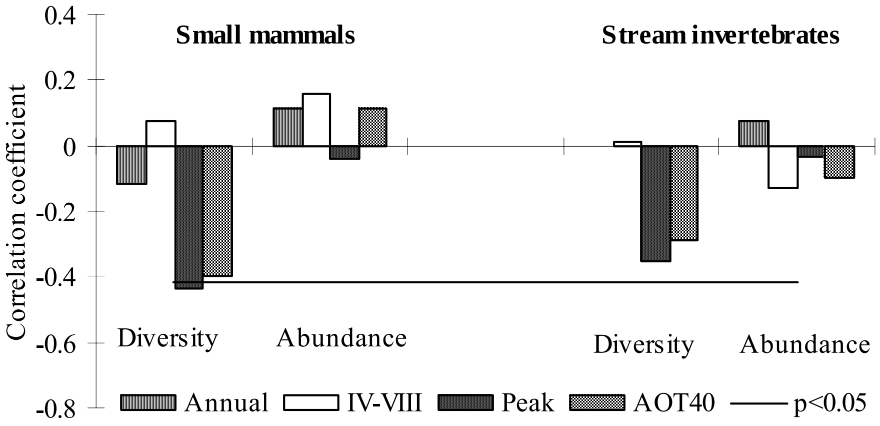

**FIGURE 4.** Relationships between ambient $O_3$ and abundance, and diversity of small mammals and stream invertebrates.

### Diversity and Abundance of Stream Macroinvertebrates

During 1994–1999, the increase in stream macroinvertebrate diversity and biomass was observed in all streams. Afterwards, until 2004, diversity estimates remained at the same level[41]. Neither mean value of $O_3$ concentration nor its peak value demonstrated statistically significant relationships with biodiversity and abundance of stream macroinvertebrates. Despite this, negative significant correlation was detected earlier and the significance varied from year to year.

## DISCUSSION

On a global scale, $O_3$ is the most widespread ambient air pollutant[4,21,52]. However, much of what we know about its effects is based on its phytotoxicity under artificial conditions[53]. A limited number of studies have examined mature forest trees under ambient $O_3$ exposure[54]. Even less is known about cause and effect relationships between $O_3$ exposure and other components of forest ecosystems, such as pedobionts and small mammals, or aquatic ecosystems, such as benthic diversity and abundance. Therefore, the presented study could be considered as an attempt to gain more insight into the relationship between the ambient $O_3$ and biota of forest ecosystems.

Tree species response to $O_3$ exposure is most likely related to differences in stomatal conductance and subsequent $O_3$ uptake[2,55]. This is why responses of plants to $O_3$ vary considerably among species[16]. Reich stated that chronic exposure to $O_3$ has a greater effect on conifers with long-lived needles than on deciduous[56]. Higher significance of the relationships between pine defoliation and $O_3$ than between birch defoliation and $O_3$ confirmed this statement. Norway spruce trees seemed to be sensitive to $O_3$ exposure in Lithuania as well[57]. *Ips typographus* often played an important negative role in the deterioration and demise of spruce ecosystems[58]. In Europe, in almost all cases, bark beetle populations occurred at locations more stressed by $O_3$[59]. Significant relationship between AOT40 for forest and spruce defoliation could be the proof of it.

Estimated changes in soil fauna biodiversity and abundance were in full agreement with the changes in tree crown defoliation[41]. Therefore, it was expected that the same $O_3$ concentrations, which resulted in changes in pine defoliation, would result in changes in biodiversity of arthropods. The relationship between the peak $O_3$ concentrations and arthropod diversity verified our assumption. In case this relationship were to be verified in future, we would be able to state that the effect of $O_3$ on trees is reinforced by the changes in arthropod structures.

The relationship between peak $O_3$ concentration and diversity of small mammals was significant as well. Most likely, it could be explained by the indirect $O_3$ effect. The changes in plant cover, frequency, or biomass and seed yield for plant species[60] — main nutrition sources — could be the key factor resulting in changes in diversity and abundance of small mammals.

The effect of ambient $O_3$ on stream invertebrates is the most subtle one. There is some suggestion that due to the effect on phytoplankton, changes in diversity of stream benthic fauna could be related to changes in ambient $O_3$[61]. The obtained data did not contradict this hypothesis.

A hierarchical approach when investigating the effect of the ambient $O_3$ concentration allowed us to verify the hypothesis that peak $O_3$ values could significantly affect various components of forest biota. Decrease in significance of peak $O_3$ value effects, in the analysis of the changes in forest biota from the most sensitive components of biota (lower life forms: plant and pedobionts) to the least sensitive (higher life form: small mammals), could be presented as proof of the causative effect of peak $O_3$ concentrations on forest ecosystems in general. Consequently, the obtained results confirmed quite well the results obtained at the Carpathian Mountain forest in Central Europe[49], where concentrations of NOx, $SO_2$ and $O_3$ were similar to the considered: (1) low levels of pollution over a long time may affect biodiversity of ecosystems[62] and (2) peak $O_3$ concentrations could be the key factors to cause them[63].

Generalizing the presented results, we express our full agreement with other authors that the $O_3$ effect may disrupt normal development of forest stands, resulting in shifts in composition, changes in genetic structure and biodiversity, and impaired ecosystem function[25]; however, detection of these effects is very subtle and elusive because of their interaction with natural stresses[64]. Uncertainties in these studies are related to complexity of the problem and cover such large areas that it is oftentimes impossible for a small group of scientists (especially one field of science) to develop meaningful results. To prevent deterioration of forest health and biodiversity successfully, well-coordinated research activities in various disciplines, such as atmospheric chemistry, forestry, botany, entomology, soil science, and dendrochronology, are recommended[7]. Therefore, in the attempt to detect possible effects of $O_3$ concentration on forest ecosystems in general, data from IMS were used. The advantage of using these data is the availability of real-time $O_3$ concentrations from monitors instead of spatially widespread measurement of $O_3$ by passive samplers.

## CONCLUSIONS

- Short periods of peak $O_3$ concentrations seem to have a significant effect on forest ecosystems, negatively affecting tree crown conditions, diversity of soil arthropods, and in some cases, small mammals and stream invertebrates.
- Decrease in significance of peak $O_3$ value effects in the analysis of changes in forest biota from the lower life forms (tree and pedobionts) to the higher life forms (small mammals) could be presented as an argument to prove causative effect of peak $O_3$ concentrations on forest ecosystems in general.
